# Variation in Parasitoid Virulence of *Tetrastichus brontispae* during the Targeting of Two Host Beetles

**DOI:** 10.3390/ijms22073581

**Published:** 2021-03-30

**Authors:** Hua-Jian Zhang, Ya-Ping Lin, Hong-Yu Li, Rui Wang, Lang Fu, Qing-Chen Jia, You-Ming Hou, Bao-Zhen Tang

**Affiliations:** 1 State Key Laboratory of Ecological Pest Control for Fujian and Taiwan Crops, Fujian Agriculture and Forestry University, Fuzhou 350002, China; huajianzhang@fafu.edu.cn (H.-J.Z.); linyaping@fafu.edu.cn (Y.-P.L.); snow_lhy@163.com (H.-Y.L.); wangrui@fafu.edu.cn (R.W.); m18305918768@163.com (L.F.); jiaqingchen@fafu.edu.cn (Q.-C.J.); 2 Key Lab of Biopesticide and Chemical Biology, Ministry of Education & Fujian Provincial Key Laboratory of Insect Ecology, Department of Plant Protection, Fujian Agriculture and Forestry University, Fuzhou 350002, China

**Keywords:** adaptive evolution, host selection, immunosuppressive, parasitic adaptability, parasitic strategy

## Abstract

In host-parasitoid interactions, antagonistic relationship drives parasitoids to vary in virulence in facing different hosts, which makes these systems excellent models for stress-induced evolutionary studies. Venom compositions varied between two strains of *Tetrastichus brontispae*, Tb-Bl and Tb-On. Tb-Bl targets *Brontispa longissima* pupae as hosts, and Tb-On is a sub-population of Tb-Bl, which has been experimentally adapted to a new host, *Octodonta nipae*. Aiming to examine variation in parasitoid virulence of the two strains toward two hosts, we used reciprocal injection experiments to compare effect of venom/ovarian fluids from the two strains on cytotoxicity, inhibition of immunity and fat body lysis of the two hosts. We found that Tb-Onvenom was more virulent towards plasmatocyte spreading, granulocyte function and phenoloxidase activity than Tb-Blvenom. Tb-Blovary was able to suppress encapsulation and phagocytosis in both hosts; however, Tb-Onovary inhibition targeted only *B. longissima*. Our data suggest that the venom undergoes rapid evolution when facing different hosts, and that the wasp has good evolutionary plasticity.

## 1. Introduction

Parasitoid wasps represent 10% to 20% of all insects, and they are the most successful and important groups of natural enemies used as biological control agents for insect pests [1,2,3]. Wasps take advantage of a variety of strategies to subdue the hosts to generate a comfortable environment for the development of their offspring, usually inducing the impairment of the host insect [4]. Endoparasitoids develop inside their hosts; thus, they must evade or counteract the specific physiological and immunological defences of their hosts [2,5,6]. Generally, in the host-parasitoid interactions, the antagonistic relationships drives endoparasitoids to evolve to become more virulent when encountering hosts with stronger selection pressure, which contributes to biological diversity and makes these systems excellent models for stress-induced evolutionary studies [2,7,8,9]. In this evolutionary race, strategies used by wasp parasitoids are mainly divided into the active and passive mechanisms. For the active mechanisms, endoparasitoids adopt a number of virulence factors, including virus-like particles (VLPs), polydnaviruses (PDVs), ovarian fluids, venom and teratocytes, to disrupt the host immune system directly or indirectly (direct intervention) and help them to escape the host encapsulation and melanisation [10,11,12,13,14,15,16]. In the case of the passive mechanisms, endoparasitoids lay their eggs in a special tissue or at a special life stage of the hosts with a low integrated immunity system to avoid encapsulation, or they depend on their inherent surface features that will mislead the host immunity to disable the recognition of the parasitoid as non-self and avoid triggering an immune induction (passive dodge) [10,17,18,19,20,21].

Parasitoid tracking of locally common host genotypes results in local adaptation [9,22]. However, upon encountering an alternative host, the parasitoid may miss an opportunity to interact with its inherent targets generated by a long-term evolution from the native host and bring about excessive energy cost [23,24,25,26,27]; thus, the variability of virulence is required. Variations of parasitoid virulence can originate from two main mechanisms; either they differ in their ability to locally evade the host immune system or they differ in their abilities to suppress the whole encapsulation response. To balance the costs in development of a higher virulence, parasitoids tend to vary in their virulence towards different hosts and do not evolve high infectivity in all hosts [28]. For example, in the *Asecodes parviclava* (Hymenoptera: Eulophidae) and *Galerucella* system, the differences in the immune defence among *G. pusilla*, *G. tenella* and *G. calmariensis* from different areas result in different virulent strains of *A. parviclava*; the wasps emerged from *G. pusilla* can favourably infect the larvae of all three species, and the wasps emerged from *G. tenella* may also successfully infect the larvae of *G. tenella* and *G. calmariensis*; nevertheless, the parasitoids from *G. calmariensis* can only infect the larvae of *G. calmariensis* [9]. Evidently, the successful parasitism of *A. parviclava* in different hosts is deeply influenced by the native host species, i.e., host species and geographic variations promote the parasitoid trade-off in virulence due to differences in the immune defence, which will finally lead to variations in the host range [9,29]. In the *Aphelinus certus* and *Aphis* system, the genetic differentiation of *Aphis* populations results in inter-population differences in parasitism, indicating that differential host specificity of the parasitoid populations is influenced by genetic distances of the hosts [30].

Variations of virulence in alternative hosts can be driven by a gene-for-gene matching, which may lead to evolution of different genes wasps allowing their adaptation to different hosts [9,29,31,32]. Notably, venom genes can evolve depending on the variation of encountered stress. For example, in the *Leptopilina boulardi* and *Drosophila* system, the injection of venom from the ISm and ISy strains to *D. melanogaster* and *D. yakuba*, respectively, may generate virulence by incapacitating the encapsulation reaction to a foreign body. In contrast, the injection of venom from the avirulent line (ISm for *D. yakuba* and ISy for *D. melanogaster*) does not have any influence on the encapsulation abilities of the hosts [33]. These results indicate that the qualitative changes in the venoms of different parasitoid lines are responsible for the variations in the parasitoid virulence [12,23,34]. Venoms contain proteins that function by altering host development [4], nutrient flux [35,36,37], immune defence [38,39,40] and lipid metabolism [41,42], and they have been confirmed to participate in multiple evolutionary processes [43,44,45], such as co-option, gene duplication, alternative splicing, multi-functionalisation, horizontal gene transfer and de novo gene synthesis [44,46,47,48]. Excitingly, in recent years, the whole venom proteomic analysis allowed the investigation of the multifunctional and evolutionary processes of the venom proteins from a comprehensive perspective and the study of the mechanisms of the evolution of countermeasures to different hosts. The gene-by-gene approach has provided additional details about the functions of the venom proteins and the interactions between the wasps and their hosts. However, studies of the parasitoid biology remain mysterious, and additional work has to be invested in the host-parasitoid interactions.

*Tetrastichus brontispae* (Eulophidae) is native to Java (Indonesia) and is a gregarious and idiobiont endoparasitoid that has been used to efficiently control two Chrysomelidae beetles, namely, *Brontispa longissima* (Gestro) and *Octodonta nipae* (Maulik), which are dangerous invasive pests of the palm plants in southern China [49,50]. *T. brontispae* has certain favourable characteristics that enhance its success to control the two beetles, including approximately 20 days for the whole lifecycle, preference for fresh host pupae, and high fecundity, with approximately 22 wasps emerging from each host [49,50]. *T. brontispae* lacks polydnavirus and virus-like filaments and thus, venom is recognised as its main virulence factor [21,51]. Proteomics and transcriptomics enable to investigate the venom compositions at the molecular level, thereby promoting the studies of the parasitoid biology of *T. brontispae* [51]. Previous comparative proteome analyses have shown that venom compositions varied between the two strains, *T. brontispae*-Bl (Tb-Bl) and *T. brontispae*-On (Tb-On), even though the major venom proteins were shared between the two strains [51]. The Tb-Bl strain targets *B. longissima* pupae as hosts, and the Tb-On strain is a derived sub-population of Tb-Bl, which has been experimentally adapted to a new host, *O. nipae*, for six years (120 generations). The functions of the venom and ovarian fluid of *T. brontispae* differ in the case of successful parasitism [21]. Therefore, in this study, the virulence of the venoms (Tb-Onvenom and Tb-Blvenom) and ovarian fluids (Tb-Onovary and Tb-Blovary) from the two strains towards the hosts were examined by reciprocal transplant style experiments aiming to explore whether the venoms and ovarian fluids from the two strains have differences in the virulence activities towards the two hosts, and confirm stress-induced evolutionary events in the parasitoid wasp. Based on previous studies on the same topic, we hypothesise that Tb-Onvenom and Tb-Blvenom will show different virulent activities in regulation host immunity, which helps *T. brontispae* generate adaptation in *B. longissima* and *O. nipae*.

## 2. Results

### 2.1. Virulence of Venom and Ovarian Fluids against Host Total Haemocyte Counts (THCs) and Differential Haemocyte Counts (DHCs)

In both un-stung hosts, the general cell composition included prohaemocytes, granulocytes, plasmatocytes, spherulocytes and oenocytoids; plasmatocytes and granulocytes were the most abundant (over 70%) (Figure S1A). Notably, granulocytes with multiple morphology were common in the two hosts (Figure S1B). THCs in *O. nipae* was substantially higher than that in *B. longissima* (24 h, *t* = 4.302, df = 56, *p* < 0.0001) (Figure S1C). After injections of PBS/venom/ovarian fluids, THCs were increased over time in both hosts, peaking at 48 h for venom/ovarian fluids and at 72 h for PBS in *B. longissima* (Figure S1D; PBS, *F*_(4, 145)_ = 4.672, *p* < 0.01; Tb-Blvenom, *F*_(4, 140)_ = 2.955, *p* < 0.05; Tb-Blovary, *F*_(4, 145)_ = 2.853, *p* < 0.05) and peaking at 96 h for all samples in *O. nipae* (Figure S1E; PBS, *F*_(4, 142)_ = 5.458, *p* < 0.001; Tb-Onvenom, *F*_(4, 147)_ = 4.871, *p* = 0.001; Tb-Onovary, *F*_(4, 152)_ = 11.66, *p* < 0.001). The injection of ovarian fluids had no influence on THCs compared with THCs detected after PBS injections in both hosts at each time point (Figure S1D,E) except that at 96 h post injection of Tb-Onovary, the THCs in *O. nipae* was significantly decreased (Figure S1F; 96 h, *F*_(2, 84)_ = 13.6, *p* < 0.0001). The injection of Tb-Onvenom induced a decrease in THCs in *O. nipae* at 24, 48 and 96 h compared with that in the PBS group (Figure S1E,F; 24 h, *F*_(2, 87)_ = 4.413, *p* < 0.05; 48 h, *F*_(2, 105)_ = 12.47, *p* < 0.0001; 96 h, *F*_(2, 84)_ = 13.6, *p* < 0.0001).

**Figure 1:**
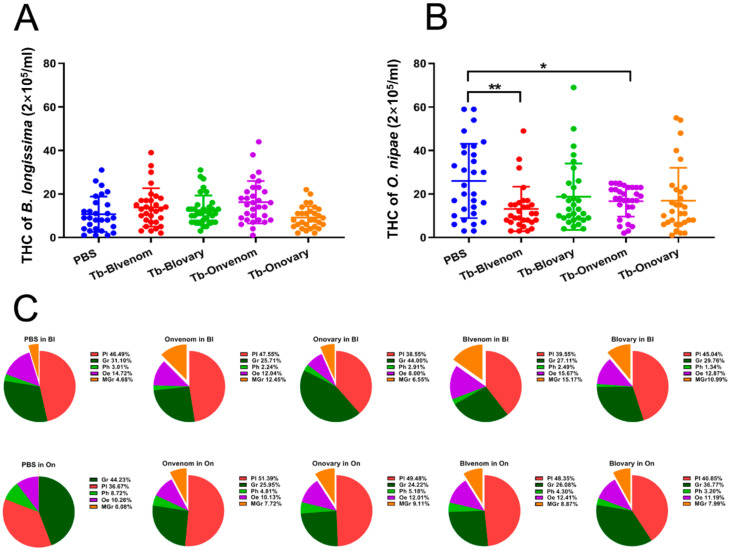
Comparison of cytotoxicity of venom or ovarian fluids injection against *O. nipae* and *B. longissima* pupae. (**A**,**B**) Total haemocyte counts (THCs) and (**C**) differential haemocyte counts (DHCs) of host pupae at 24 h after injection with venom or ovarian fluids. * and ** represent a significant difference at the *p* < 0.05 level and *p* < 0.01 level, respectively (Welch’s/Brown-Forsythe ANOVA followed by Dunnett’s T3 test).

To investigate whether mother’s host experience influenced the virulence of venom and ovarian fluids, Tb-Onvenom and Tb-Onovary were injected into *B. longissima*, and Tb-Blvenom and Tb-Blovary were injected into *O. nipae*. The reciprocal virulent influence was evaluated at 24 h post injection. The results indicate that in *B. longissima* pupae, the injection of Tb-Onvenom, Tb-Onovary, Tb-Blvenom and Tb-Blovary had no influence on THCs, while in *O. nipae* pupae, the injection of Tb-Onvenom or Tb-Blvenom induced a significant decrease in THCs (*F*_(4, 116_._6)_ = 3.710, *p* < 0.01; *W*_(4, 70.05)_ = 3.139, *p* = 0.0196) (Figure 1A,B). In the case of DHCs, the ratio of plasmatocytes to granulocytes was approximately 1:1.2 in *O. nipae* and 1.5:1 in *B. longissima* (Figure 1C), respectively, after PBS injection. In *B. longissima*, macrogranulocytes were significantly increased after challenge by virulent factors (Figure 1C), especially by Tb-Onvenom (12.45%) and Tb-Blvenom (15.7%), compared to 4.68% detected in the case of PBS (*χ*^2^ = 62.255, df = 16, *p* < 0.0001). In *O. nipae*, the injection of venom or ovarian fluids from both strains markedly influenced DHCs (Figure 1C; *χ*^2^ = 128.538, df = 16, *p* < 0.0001) and induced a decrease in granulocytes by 19% (dark green section), an increase in plasmatocytes by 15% (pink section) and an increase in macrogranulocytes by 8%.

### 2.2. Virulence of Venom and Ovarian Fluids against Host Haemocyte Spreading

**Figure 2:**
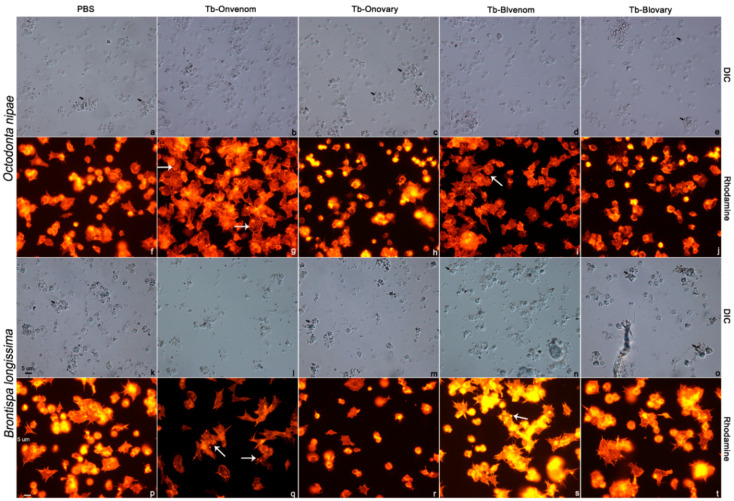
Haemocyte spreading assay in *O. nipae* and *B. longissima* pupae after the injection with virulent factors. (**a**–**e**,**k**–**o**) haemocytes visualised by differential interference microscopy; (**f**–**j**,**p**–**t**) fluorescence of rhodamine phalloidin-labelled cytoskeleton visualised by fluorescence microscopy. Star-like clusters in venom injection groups are marked by white arrows. Aggregates are marked by thick black arrows. Scale bars, 5 μm.

Haemocyte spreading is recognised as a key indicator of cell viability. In this assay, rhodamine-phalloidin with red fluorescence was used to visualise the degrees of haemocyte extension after various treatments. The results indicate that in *O. nipae* pupae injected with PBS, haemocytes were highly organised and whipcord (Figure 2a,f). After the injection of venoms from the both wasp strains, plasmatocytes were extensively spread and extended pseudopods; the majority of the plasmatocytes were friable and had irregular polygonal morphologies (Figure 2b,d,g,i). Moreover, star-like F-actin clusters formed by the aggregates of actin cytoskeleton were observed all over the target plasmatocytes (Figure 2g,i). Notably, in the groups injected with the venom, the populations of granulocytes and macrogranulocytes were decreased (Figure 2b,d,g,i), and only a few completely intact macrogranulocytes and abundant free granules were observed compared to those in the groups injected with PBS or ovarian fluids (Figure 2a,c,e,f,h,j). Ovarian fluids had little effect on haemocytes (Figure 2c,e,h,j).

In *B. longissima* pupae, haemocytes in PBS treatment showed similar morphology to that in *O. nipae* pupae (Figure 2k,p). Ovarian fluids had little influences on haemocyte spreading (Figure 2m,o,r,t). The effects of venom injection on haemocyte spreading were similar to that in *O. nipae* pupae (Figure 2g,l,n,s); Tb-Onvenom had stronger effect on macrogranulocyte destruction than that observed in the case of Tb-Blvenom (Figure 2i,n,q,s).

### 2.3. Differential Virulence of Venom and Ovarian Fluids against Host Phagocytosis

**Figure 3:**
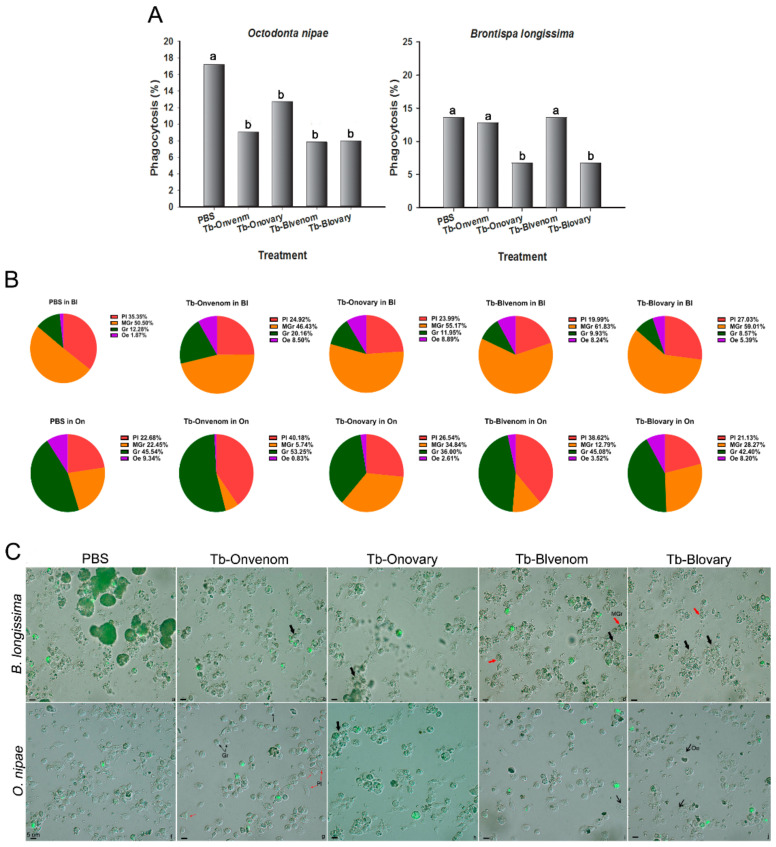
Haemocyte phagocytosis assay in *O. nipae* and *B. longissima* pupae after the injection with virulent factors. *O. nipae* or *B. longissima* pupae were pre-injected with virulent factors for 12 h followed by injections with heat-inactivated FITC-labelled *E. coli* for another 12 h. Pupae injected with PBS were used as a control group. (**A**) Phagocytosis rate after treatments. Different lowercase letters represent a significant difference at the *p* < 0.001 level (R × C *chi*-square test). (**B**) DHCs of *O. nipae* and *B. longissima* pupae injected with virulent factors and FITC-labelled *E. coli*. Pl, plasmatocyte (red); Gr, granulocyte (green); MGr, macrogranulocyte (orange); Oe, oenocytoid (purple). (**C**) Haemocytes visualised by differential interference microscopy and fluorescence images. Green fluorescence corresponds to FITC-labelled *E. coli*. Aggregates and macrogranulocytes are marked with thick black and red arrows, respectively. Pl and Gr are marked with thin red and black arrows, respectively. Scale bars, 5 μm.

The virulence of venom or ovarian fluids against host phagocytosis was measured as the number of phagocytised FITC-labelled *E. coli*. In *O. nipae* pupae, the number of bacteria engulfed by haemocytes was significantly reduced after injection with venom or Tb-Blovary, and the percentage of phagocytosis was approximately 48% of that observed in the PBS group (Figure 3A left panel; *χ*^2^ = 99.553, df = 4, *p* < 0.0001). In *B. longissima* pupae, the venom from the both strains did not influence the phagocytosis; however, ovarian fluids from the both strains significantly inhibited the host phagocytosis by 48% (Figure 3A right panel; *χ*^2^ = 74.748, df = 4, *p* < 0.0001).

Notably, the percentage of macrogranulocytes plus granulocytes in *B. longissima* (50.50% + 12.28%) and *O. nipae* (22.45% + 45.54%) was increased after a challenge with *E. coli* compared to that after challenge with PBS (Figure 3B; *B. longissima*, *χ*^2^ = 198.116, df = 3, *p* < 0.0001; *O. nipae*, *χ*^2^ = 86.652, df = 3, *p* < 0.0001. In the case of PBS, please see Figure 1C: *B. longissima* (4.68% + 31.10%), *O. nipae* (0.08% + 36.67%)). In *B. longissima*, after the injection with venom or ovarian fluids, an *E. coli* challenge had little influence on the haemocyte composition (Figure 3B upper panel; *χ*^2^ = 7.604, df = 12, *p* = 0.107). In *O. nipae* pupae after the injection with ovarian fluids, the percentage of macrogranulocytes in the *E. coli*-challenged pupae was continuously increasing; however, in the venom-injected groups, higher number of plasmatocytes and lower number of macrogranulocytes were observed (Figure 3B down panel; χ^2^ = 871.360, df = 12, *p* < 0.0001). Interestingly, venom induced individual haemocyte scattering, and ovarian fluids induced haemocyte aggregation in *O. nipae*; however, in *B. longissima*, the venom and ovarian fluids induced haemocyte aggregation (Figure 3C). These results suggest that granulocytes are dominant in phagocytosis and aggregation (Figure 3C) and that macrogranulocytes are the main force of cellular immunity and are susceptible to the pathogen (Figure 3B).

### 2.4. Differential Virulence of Venom and Ovarian Fluids against B. Longissima and O. nipae Encapsulation

**Figure 4:**
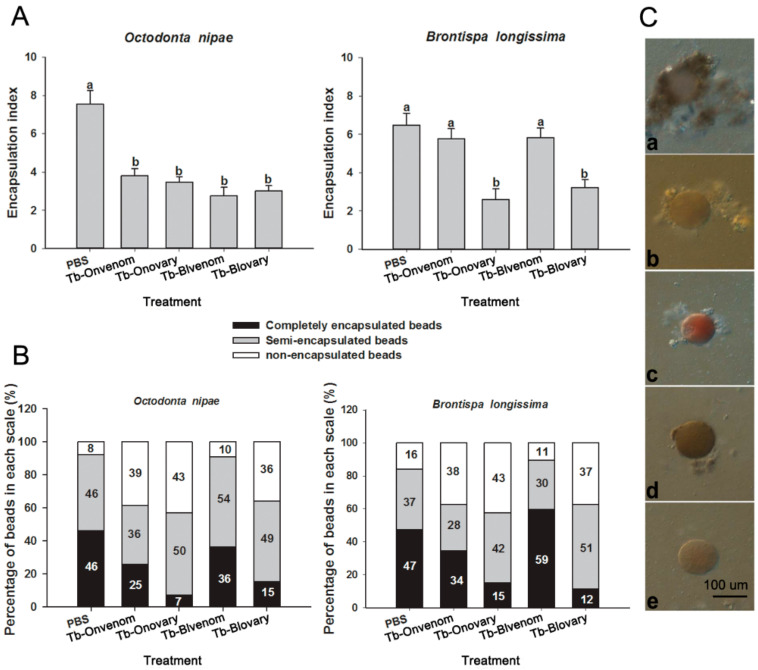
Effect of various virulent factors on haemocyte encapsulation. (**A**) Encapsulation index of *O. nipae* and *B. longissima* haemocytes at 24 h after treatments. (**B**) Number of beads of each scale in various treatments. The encapsulated beads were separated into three scales, depending on the number of haemocytes attached to the beads. (**C**) Beads with varying degrees of encapsulation. Different letters correspond to significant differences at the 0.05 level between different tissues (one-way ANOVA followed by Dunnett’s test). Scale bars, 100 μm.

Sephadex A-50 beads of equal diameter were selected to evaluate the encapsulation rate. In *O. nipae* pupae, the venom or ovarian fluids from both wasp strains induced an effective inhibition (approximately 55%) of host haemocyte encapsulation (Figure 4A–C; *F*_(4, 20)_ = 18.665, *p* < 0.001). In *B. longissima* pupae, ovarian fluids from both wasp strains induced a decrease in host encapsulation index (EI) by 50%, while the venom did not influence the host encapsulation (Figure 4A–C; *F*_(4, 20)_ = 14.733, *p* < 0.001).

### 2.5. Differential Virulence of Venom and Ovarian Fluids against Host PO Activity

**Figure 5:**
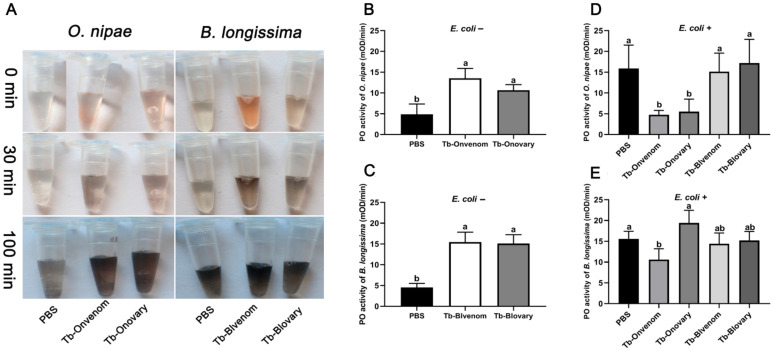
Effect of virulent factors on phenoloxidase (PO) activity 24 h post injection. (**A**) Melanisation of *O. nipae* and *B. longissima* haemolymph after the injection with venom or ovarian fluids. (**B**,**C**) PO activity of *O. nipae* or *B. longissima* haemolymph after venom and ovarian fluids injection. (**D**,**E**) PO activity of haemolymph collected from *O. nipae* or *B. longissima* pupae after the injection with various virulent factors and incubated with heat-inactivated *E. coli* for 10 min. Average *V*_max_ was used to evaluate PO activity. Error bars indicate standard deviations of the mean of five independent biological replications. Different letters correspond to significant differences at the 0.05 level between different tissues (one-way ANOVA followed by Dunnett’s test).

The activation of pro-PO (PPO) is an important part of humoural immunity. Interestingly, the same volume of haemolymph had substantially higher PO activity in *B. longissima* than that in *O. nipae* (Figure S2; *t* = 7.880, df = 13, *p* < 0.0001). After the injection of the venom or ovarian fluids into *B. longissima* or *O. nipae* pupae, the haemolymph spontaneously became black within 100 min (Figure 5A), and the PO activity of the haemolymph was increased by 2–3 times (Figure 5B,C; On, *F*_(2, 10)_ = 16.74, *p* < 0.001; Bl, *F*_(2, 11)_ = 52.69, *p* < 0.0001); however, the colour of the haemolymph in the PBS group was only slightly changed within 100 min (Figure 5A). Usually, PO activity in the haemolymph can be induced by microbial elicitors, such as *E. coli*. However, in *O. nipae* pupae injected with Tb-Onvenom or Tb-Onovary, the addition of *E. coli* to the haemolymph did not induce an increase in PO activity (Figure 5D). Similarly, the addition of *E. coli* did not induce an increase in PO activity in *B. longissima* pupae injected with Tb-Onvenom. However, in Tb-Blvenom- or Tb-Blovary-injected host pupae (in *B. longissima* and *O. nipae*), the PO activity was still induced by *E. coli* (Figure 5D,E; On, *F*_(4, 16)_ = 8.592, *p* < 0.001; Bl, *F*_(4, 20)_ = 7.984, *p* < 0.001).

### 2.6. Venom Promotes B. Longissima and O. Nipae Fat Body Lysis

**Figure 6:**
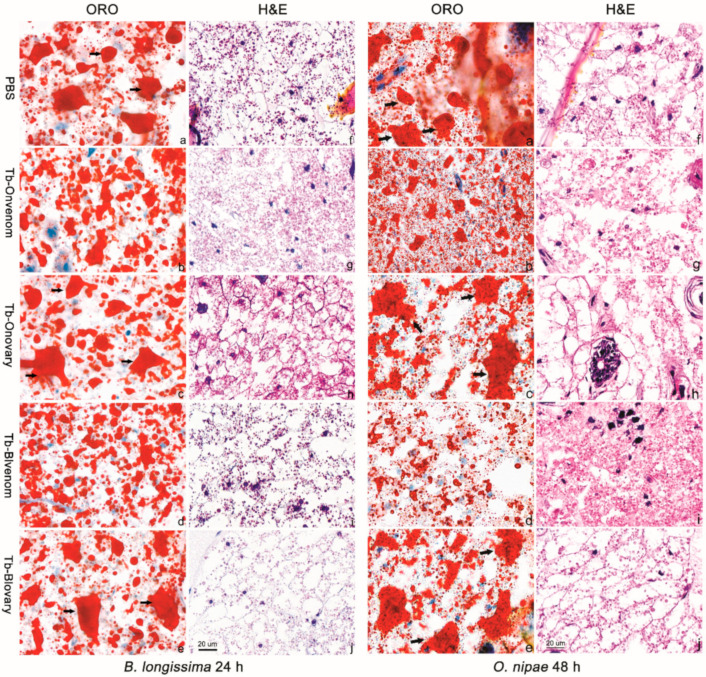
Effect of virulent factors on *B. longissima* and *O. nipae* pupae fat body lysis at 24 h and 48 h post injection, respectively. ORO and H&E stained fat body tissues of *B. longissima* and *O. nipae* pupae after the injection with PBS (**a**,**f**), Tb-Onvenom (**b**,**g**), Tb-Onovary (**c**,**h**), Tb-Blvenom (**d**,**i**) or Tb-Blovary (**e**,**j**); original magnification, 40×. Larger pieces of fat body are marked by thick black arrows in the ORO sections. Scale bar, 20 μm.

Degradation of the host fat body was determined by ORO and H&E staining. In *O. nipae*, the fat body began to lyse 24 h after the injection with venom (Figure S3g,i), and was severely degraded and decomposed at 48 h (Figure 6b,d,g,i). Tb-Blvenom caused a stronger effect on fat body lysis compared to that observed in the case of Tb-Onvenom (Figure 6b,d,g,i). In detail, venom induced fat body decomposition into small pieces, resulting in appearance of abundant lipid droplets (Figure 6b,d) and severe degradation of adipose cell (Figure 6g,i). After injection with PBS or ovarian fluids, a high number of large fragments of fat body remained (black arrow, Figure 6a,c,e), and adipose cells were intact and tightly arranged (Figure 6f,h,j). In *B. longissima,* 24 h after the injection with the venom, the fat body was severely decomposed and degraded, and a more pronounced effect was caused by Tb-Blvenom (Figure 6b,d,g,i). Notably, the fat body in *B. longissima* pupae appeared to be easily degraded even in the PBS or ovarian fluid-injected groups, thus making it difficult to evaluate suitable sections (Figure 6 and Figure S4).

### 2.7. Comparison of Transcriptome Analysis between O. Nipae and B. Longissima

**Figure 7:**
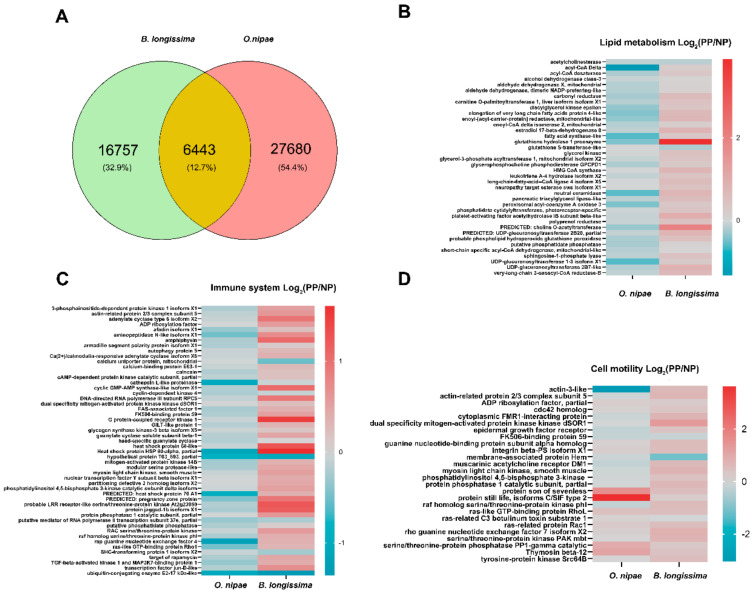
Comparison of transcriptomic analysis of *B. longissima* and *O. nipae*. (**A**) Homology analysis of *O. nipae* and *B. longissima* unigenes with a 70% coverage and an E-value cut-off of 10^−5^; (**B**–**D**) Differential expression analysis of lipid metabolism-related (**B**), immune system-related (**C**), and cell motility-related (**D**) homology genes in *O. nipae* and *B. longissima* between non-parasitised (NP) and parasitised pupae (PP).

Comparative transcriptome analysis was used to visualise the differentiation of physiological dimension between *O. nipae* and *B. longissima.* A pair of genes with a 70% coverage and an E-value cut-off of 10^−5^ was considered to be a homologous gene between the two hosts. The homologous comparison results indicate that only 6443 genes were conserved, and 83.18% and 73.31% unigenes were unique for *O. nipae* and *B. longissima*, respectively (Figure 7A, Figure S5 and Table S1).

The transcriptome profiles of *B. longissima* and *O. nipae* pupae were significantly influenced 24 h after the infection with *T. brontispae*. Using the criteria of log_2_-fold > 1.5 and FDR < 0.05, 706 unigenes (577 upregulated and 129 downregulated) were significantly differentially expressed in *O. nipae* (Figure S6); in *B. longissima*, the majority of the differentially expressed genes were upregulated (242 upregulated and 1 downregulated, Figure S6). For an additional focus on the conserved genes related to lipid metabolism, immune system and cell motility, we performed a comparative expression profile for these genes in samples prepared after the infection by *T. brontispae*. The results indicate that the majority of the genes in *O. nipae* are downregulated or unchanged after parasitism, while in *B. longissima*, the genes were upregulated (Figure 7B–D).

## 3. Discussion

Venom genes encode for multiple factors to target the humoural and cellular immune systems and nutritional metabolism of the host to help parasitoid offspring avoid the killing activity of host defence and gain enough energy supply [52,53,54,55]. In the present study, venom induced cytotoxicity in haemocytes, including a decline in THCs, a reduction in granulocyte percentage and an increase in percentage of plasmatocytes and macrogranulocytes in *O. nipae* pupae. Similarly, in the *Pimpla turionellae*-*Galleria mellonella* system, venom injection induced a reduction in the number of granulocytes and an increase in the number of plasmatocytes; more importantly, such injection induced the formation of vacuoles within the cytoplasm in granulocytes with eventual death of granulocytes [29,56]. Macrogranulocytes are activated granulocytes, which are characterised by large cytoplasmic vacuoles, undergo subsequent apoptosis and eventually discharge a large amount of materials [57,58,59]. In our system, an increase in percentage of macrogranulocytes was detected, and we thus hypothesise that the *T. brontispae* venom induces the unloading of macrogranulocytes (weapons-granulocytes), that the transformation of granulocytes into macrogranulocytes without a supplement results in a decrease in granulocytes, and subsequently, that macrogranulocytes undergo apoptosis leading to a decrease in THCs.

Additionally, venom can significantly inhibit the spreading of plasmatocytes for a number of reasons. The inhibition of F-actin development within haemocytes is universally associated with disturbance of actin cytoskeleton [60]. Cytoskeleton rearrangements and microfilaments are involved in encapsulation and phagocytosis [61,62]; hence, the formation of star-like actin clusters (the actin aggregates) in plasmatocytes in the venom injection group in the present study most likely caused the inhibition of haemocyte spreading, encapsulation and phagocytosis. Similarly, the concerted action of two toxins, TccC3 and TccC5 adenosine diphosphate-ribosyltransferases from *Photorhabdus luminescens*, induced the clustering of F-actin in *Galleria mellonella* haemocytes, thus inhibiting phagocytosis of the target haemocytes [63].

PPO is synthesised in the haemocytes and is released into the haemolymph due to the rupture of haemocytes [64,65]. PPO is usually synthesised in oenocytoids [66]. Recently, it has been demonstrated that PPO can also be produced in plasmatocytes (lamellocytes) and granulocytes. In *Drosophila*, PPO3 produced by lamellocytes emerged as a novel defence mechanism against parasitoid wasps [67]. PO activity was determined in granulocytes in *Mythimna separata* [68]. A previous study demonstrated that in *O. nipae*, in addition to oenocytoids, granulocytes were PPO/PO-positive haemocytes [69]. Granulocytes from *O. nipae* pupae treated with Triton X−100 can be stained with an anti-PPO antibody to produce a strong fluorescence signal (Zhang et al., unpublished data). Thus, we hypothesise that in *O. nipae*, granulocytes can also produce PPO. After the injection of venoms or ovarian fluids, the percentage of oenocytoids did not change; hence, an increase in the PO activity may be attributed to the apoptosis of macrogranulocytes rather than to the rupture of oenocytoids. Moreover, the apoptosis of macrogranulocytes and the subsequent release of their inclusions is related to fat body decomposition and degradation in *Helicoverpa armigera* [59]. This mechanism may be involved in induction of the lysis of host fat body by venom of *T. brontispae*.

Antagonistic interactions between parasitoids and their hosts drive evolution and generate diversity leading to coevolution of both species [8]. Trade-offs caused by strength of selection in the host-parasitoid systems are characterised by the host resistance strategies and parasitoid virulence tactics [9]. In our system, evolutionary events occur only in the parasitoid. Venoms from Tb-On and Tb-Bl displayed virulent activities towards the two hosts, and Tb-Onvenom was more virulent towards plasmatocyte spreading, granulocyte function and PO activity than Tb-Blvenom, suggesting a variability in the virulence of *T. brontispae* venom under *O. nipae* selection. In the case of ovarian fluids with complex function, some of these functions may be complementary to the effects of venom [70], others may act as an antidote to reverse the effects of the venom [71], and some may function independently [72]. Tb-Blovary was able to suppress encapsulation and phagocytosis of *E. coli* in both hosts; however, Tb-Onovary inhibition targeted only *B. longissima* Thus, both venom and ovarian fluids contribute to the inhibition of host immunity by targeting *B. longissima* pupae hosts; however, the contribution of venom to the inhibition of *O. nipe* immunity is higher than the impact of ovarian fluid. Regardless of the host, venoms from both strains were virulent in host fat body lysis. Overall, these results indicate that the evolution of venoms and ovarian fluids in *T. brontispae* occurs in response to host selection.

Venom compositions in parasitoid wasps, which are important counter-resistance traits, are undergoing rapid evolution while facing various hosts, suggesting a potential for adaptation to new hosts [45]. In the case of *L. boulardi*, variation in the venom counter-resistance in facing *D. yakuba* is correlated to variability of the effects on the PO cascade and plasmatocyte number; in *D. melanogaster*, this phenomenon is related to the effects on the lamellocyte number and morphology [38,73,74]. Multiple inheritance systems transfer accommodation and channel stress-induced variability between the generations drive long-term persistence and stress-induced evolutionary adaptations [75]. It seems reasonable that *T. brontispae* generates evolutionary adaptations in *B. longissima* and *O. nipae.* Substantial differences in physiological dimension between *B. longissima* and *O. nipae* indicated by comparative transcriptome analysis induce different types of stress, such as immune defence, energy supply and development time and space, in *T. brontispae*. This is the reason why *T. brontispae* has to adjust when targeting *O. nipae*. The stronger disruptive effect of Tb-Onvenom on plasmatocyte spreading, granulocyte function and PO activity may be due to the stronger selection stress of cellular immunity from *O. nipae.*

Local adaptation can be a major cause of evolution and speciation [30,76]. In *T. brontispae- B. longissima/O. nipae* system, Tb-On or Tb-Bl did not demonstrate differences in parasitism rate, emergence rate and sex ratio towards *O. nipae* and *B. longissima* (Lin et al., unpublished data). These indicate that Tb-On or Tb-Bl could also perform well when encountering non-local host (the non-local host of Tb-On is *B. longissima*, and the non-local host of Tb-Bl is *O. nipae*). Thus, this wasp has ample evolutionary plasticity, and the theory of local adaptation does not apply to this system because we have shown that the virulence of the venom is enhanced under the pressure of *O. nipae*-driven selection. Similarly, in the *Cotesia melitaearum* and *Melitaea cinxia* system, no local adaptation was found in different populations; however, there was a local genetic variation on a short-term scale for hundreds of generations [77]. In the *Lysiphlebus fabarum* and *Aphis fabae* system, variations in the virulence of parasitoids within four different populations was observed; however, the lack of genetic differentiation or genotype-specific interactions suggested a lack of local adaptation, regardless of genetic variation [78]. The comparison of susceptibility of *Drosophila melanogaster* and virulence of *Asobara tabida* indicates that local adaptation was not detected across European populations [74]. Thus, local adaptation may exist in the system in which the parasitoid performed various adjustments to host populations, similar to the *Drosophila* and *L. boulardi* system [79] and the *Galerucella* and *A. parviclava* system [9].

## 4. Materials and Methods

### 4.1. Hosts and Wasp Strains

*Brontispa longissima* and *O. nipae* were reared under the conditions of 75 ± 5% relative humidity, 25 ± 1 °C and a photoperiod of 12:12 (light: dark), as described previously [80], and they were supplied with fresh leaves of Canary Island date palm, *Phoenix canariensis* Hort. Ex Chabaud, as a diet. The origins of *T. brontispae* and two strains of the wasps were as described previously [50,51]. Briefly, *T. brontispae*-Bl (Tb-Bl) was cultivated with one-day-old (newly exuviated) *B. longissima* pupae, and *T. brontispae*-On (Tb-On), a derived sub-population of Tb-Bl, underwent a host transposition from *B. longissima* to *O. nipae* consecutively for six years (120 generations). Wasp strains were maintained under the same conditions as the hosts. After emergence, adult wasps were fed on a streak of 10% sucrose solution in plastic containers as described previously [50]. Because female wasps have the highest rate of parasitism on the newly exuviated host pupae, one-day-old host pupae were selected for all subsequent experiments unless specified otherwise.

### 4.2. Preparation of Venom and Ovarian Fluids from the Two Stains

Female adult wasps without parasitism experience of each strain were selected for collection of the venom and ovarian fluids 24–48 h after emergence. The female wasps were dissected on ice to separate the venom apparatus and ovary, as described previously [21]. Approximately 200 venom apparatuses or ovaries from each strain were combined in 40 µL PBS (0.01 M, 8 g/L NaCl, 0.20 g/L KCl, 1.44 g/L Na_2_HPO_4_ and 0.24 g/L KH_2_PO_4_, pH 7.4) in a PCR tube (5 equivalents per µL). Then, the venom apparatuses from Tb-On and Tb-Bl were homogenised by a tissue grinder and centrifuged at 12,000 g for 10 min at 4 °C to collect the supernatant, after which the samples were labelled as Tb-Onvenom and Tb-Blvenom, respectively; ovaries from Tb-On and Tb-Bl were dissected and centrifuged at 10,000 g for 10 min at 4 °C to remove eggs and cellular debris, and the resulting supernatants were labelled as Tb-Onovary and Tb-Blovary, respectively. All prepared samples were divided into small aliquots in a PCR tube and stored at −80 °C until use.

### 4.3. Assay of Total Haemocyte Counts (THCs) and Differential Haemocyte Counts (DHCs)

To test the virulence of *T. brontispae* of the Tb-Bl and Tb-On strains on its respective host haemocytes, 207 nl of Tb-Onvenom or Tb-Onovary (about one equivalent to each host) was injected into *O. nipae* pupae, and the same volume of Tb-Blvenom or Tb-Blovary was injected into *B. longissima* pupae by a NANOLITER 2010 (WPI, Sarasota, FL, USA). PBS injection was set as a control group for the corresponding host. One microliter of host haemolymph was harvested from each treatment at various time points (12, 24, 48, 72 and 96 h) after the injection. The haemolymph (one microliter) was then diluted with four microliters of Schneider’s *Drosophila* medium (Thermo Fisher, Waltham, MA, USA) and subsequently loaded on a haemocytometer (Shanghai Qiujing Corporation, Shanghai, China) for THC and DHC analyses; the samples were imaged and analysed by fluorescence and differential interference contrast microscopy with the NIS-N-viewer software (Nikon Ni-U, Tokyo, Japan). Each treatment at each time point was performed in at least 30 replicates.

To test whether the changes in the quality and/or quantity in the venoms and ovarian fluids of the two strains are responsible for the variations in parasitoid virulence, Tb-Onvenom or Tb-Onovary was injected into *B. longissima* pupae and Tb-Blvenom or Tb-Blovary was reciprocally injected into *O. nipae* pupae. Because the virulence of the wasp had the visible effects (e.g., THCs and DHCs) on the host 24 h after the injection of the venom or ovarian fluids, host pupae were collected 24 h after the injection in all subsequent experiments unless specified otherwise.

### 4.4. In Vitro Haemocyte Spreading Assay

In this assay, 3 μL of the haemolymph drawn from five pupae of *O. nipae* at 24 h post-injection with 207 nl of Tb-Onvenom, Tb-Onovary, Tb-Blvenom, Tb-Blovary or PBS was mixed with 2 μL physiological saline solution (PS) in each treatment; in the case of *B. longissimi*, a 5-μL haemolymph sample from five injected pupae was not diluted with PS. The collected haemolymph was incubated at room temperature for 30 min on a lysine-coated glass (CITOGLAS^®^ SHIATI^®^, China) in a wet black box and the glass was washed. The attached haemocytes were fixed by 4% paraformaldehyde (diluted with PBS) for 15 min and washed with PBS. The haemocytes were then treated with 0.1% Triton X-100 (diluted with PBS) for 5 min, washed and incubated with 20 μL 1% BSA for 1 h in a wet black box. Then, 10 μL 10% rhodamine phalloidin (diluted in PBS) (Thermo Fisher) was added to target F-actin for 30 min, and the samples were washed. Subsequently, 10 μL 1 μg/μL DAPI (4′,6-diamidino-2-phenylindole 4′,6-diamidino-2-phenylindole) was added to stain the nuclei for 10 min. The treated haemocytes were observed in the blue and red channels to detect fluorescence of the nuclei and cytoskeleton, respectively, by fluorescence and differential interference contrast microscopy. Each treatment included four biological replicates, and at least 2000 haemocytes were counted.

### 4.5. In Vivo Phagocytosis Assay

Approximately 138 nl of heat-inactivated Fluorescein isothiocyanate (FITC)-labelled *E. coli* (OD_600_ = 0.5) was injected into each host pupa (*B. longissima* or *O. nipae*) that was pre-injected with 207 nl of Tb-Onvenom, Tb-Onovary, Tb-Blvenom, Tb-Blovary or PBS for 12 h (a total of ten treatments), and the treated pupae were incubated for another 12 h. For *O. nipae* pupae, 3 μL of the haemolymph collected from five pupae was mixed with 2 μL PS; for *B. longissima*, 5 μL of the haemolymph collected from five pupae was not diluted with PS. Then, the haemolymph was dropped onto a lysine-coated glass and incubated for 30 min at room temperature in a wet black box. After the incubation, the haemocytes were washed three times with PBS and then observed under a fluorescence and differential interference contrast microscope. The images and the phagocytosis rate were analysed with the NIS-N-viewer software. Each treatment included four biological replicates, and at least 2000 haemocytes were counted.

### 4.6. In Vitro Haemocyte Encapsulation Assay

Approximately 207 nl of Tb-Onvenom or Tb-Onovary was injected into *O. nipae* and *B. longissima* pupae; similarly, the same volume of Tb-Blvenom or Tb-Blovary was injected into *O. nipae* and *B. longissima* pupae. PBS injections into *O. nipae* and *B. longissima* pupae were used as the control groups. At 24 h post-injection, five microliters of the haemolymph was harvested from at least five pupae in each treatment and mixed with five microliters of Schneider’s *Drosophila* medium that contained ten dextran gel beads (DEAE-Sephadex A-50; beads of equal diameter were selected for Congo red staining). The mixture was incubated at room temperature for 24 h on a tube revolver (TR-02U, Crystal Industry, Dallas, TX, USA) and rotated at a speed of 10 rpm; then, the encapsulated or unencapsulated beads were observed under a Nikon differential interference contrast microscope and imaged with the NIS-Elements D 4.30.00 software. The encapsulation index (EI) was quantified as the thickness (T) and area (A) of haemocytes attached to the beads according to the previously described method [81]. Each treatment was performed in at least five replicates.

### 4.7. In Vitro Haemolymph Phenoloxidase (PO) Activity Assay

Approximately 2.5 μL of the haemolymph from five *B. longissima* or *O. nipae* pupae at 24 h post-injection with 207 nl Tb-Onvenom, Tb-Onovary, Tb-Blvenom, Tb-Blovary or PBS was mixed with 7.5 μL Schneider’s *Drosophila* medium containing heat-inactivated *E. coli* as stimulus and incubated at room temperature for 10 min; then, PBS was added to a final volume of 70 μL. The PO activity was assayed as described previously [82,83]. In brief, for each sample, 20 μL pretreated haemolymph was mixed with 30 μL PBS in a 96 well microtiter plate (JingAn Biological, Shanghai, China); then, 100 μL L-3,4-dihydroxyphenylalanine (L-DOPA, 8 mg/mL, dissolved in PBS) was added to each well to a final volume of 150 μL. PO activity was measured by a spectrophotometer (SpectraMax, Molecular Devices Corporation, USA) with the following settings: absorbance, 490 nm; intervals time, 60 s; total reaction time, 30 min; temperature, 28 °C. Three technical replicates were used for each assay to obtain an average *V*_max_ (the slope value of the reaction curve) to assay the PO activity. For each treatment, PO activity was averaged from five biological replicates.

### 4.8. Virulence of the Venom and Ovarian Fluids on Host Fat Body Lysis

Haematoxylin-eosin (H&E) and Oil Red O (ORO) staining were used to measure the virulence of the venom and ovarian fluids on host fat body lysis. The *B. longissima* or *O. nipae* pupae at 24 h post-injection with 207 nl Tb-Onvenom, Tb-Onovary, Tb-Blvenom, Tb-Blovary or PBS were pierced at the appendage with a small insect needle and infiltrated in 4% paraformaldehyde for 2 h. The treated pupae were shipped to Wuhan Servicebio Technology Co., Ltd., Wuhan, China, for microscopic observation. Each pupa was fixed with SAKURA Tissue-Tek^®^ O.C.T. compound (SAKURA, Tokyo, Japan) and sectioned at a thickness of 10 μm (Cryostar NX70, Thermo Scientific, Waltham, MA, USA). The ultrathin sections were mounted on glass slides and subjected to ORO staining for evaluation of dispersion of lipid droplets [84,85]. For H&E staining, 4% paraformaldehyde-fixed pupae were dehydrated in a gradient of alcohol, embedded in paraffin (Taikang, TKY-BMB, Wuhan, Hubei, China) and sectioned at 4 μm of thickness by a freezing microtome (SLEE, MNT, Mainz, Germany). Paraffin-embedded pupae sections were dewaxed, rehydrated, and stained with H&E (C0105, Solarbio Science & Technology, Beijing, China). Then, xylene was used to dehydrate and clear the sections, and the coverslips were mounted with synthetic media (Permount, Zhanyun, Shanghai, China). The sections were analysed and the images were recorded using a biological microscope (Olympus, CX43, Shinjuku-ku, Tokyo, Japan); images were processed by the Caseviewer software.

### 4.9. Analyses of Host Transcriptomics

One-day-old *B. longissima* and *O. nipae* pupae were infected by Tb-Bl and Tb-On, respectively. Pupae were harvested at 24 h post-parasitisation and non-parasitised pupae were synchronously collected as controls. A total of four libraries were constructed, including Bl-NP, Bl-PP, On-NP and On-PP. For each library, three biological replicates were required and at least seven pupae were included in each biological replicate. Total RNA samples were isolated by an Eastep^®^ Super total RNA extraction kit (Promega, Fitchburg, WI, USA) according to the manufacturer’s protocol and sent to Majorbio^®^ company (Shanghai, China) for sequencing and analysis. The construction and sequencing of a total of four cDNA libraries was implemented on an Illumina Hiseq 2500 platform with paired-end reads of 125 bp. Subsequent analysis of the transcriptomes was performed similar to the previous protocols [86]. In brief, the clean reads were assembled using Trinity [87] and output as unigenes. Unigenes were then annotated by BLASTx with an E-value ≤ 1e^−5^ against the NCBI non-redundant (NR), Swiss-Prot, Cluster of Orthologous Groups (COG), Gene ontology (GO) and Kyoto Encyclopedia of Genes and Genome (KEGG) protein databases. Protein domains were annotated with the Pfam 26.0 database (November 2011, 13,672 families) using the PfamScan software [88]. The relative abundance of unigenes was output as TPM (Transcripts Per Million reads) values by RSEM software. Differentially expressed genes (DEGs) between non-parasitised and parasitised host pupae were identified on the basis of log_2_fold ≥ 0.585 (fold change ≥ 1.5) and *q*-value < 0.05 (corrected *p*-value with Benjamini/Hochberg test using DESeq2), and then subjected to GO functional and KEGG pathway enrichment analyses using Fisher’s exact test. Only GO terms or biological pathways with the adjusted *p*-value < 0.05 was considered as statistically significantly enriched.

For orthologue analysis in two species, *O. nipae* unigenes with high-quality were applied to construct a local blast database and *B. longissima* unigenes were utilised for blast alignment with 70% coverage, 70% identity and E-value ≤ 1e^−5^. In turn, *B. longissima* unigenes were set as a local blast database and *O. nipae* unigenes were used to do blast alignment with the same parameter settings. Finally, *O. nipae* unique genes and *B. longissima* unique genes were subjected to GO and KEGG function analyses.

### 4.10. Statistical Analysis

One-way ANOVA and Tukey’s multiple comparisons test (*p* < 0.05) was performed to analyse the data of variation of host THCs at different time points after injection of virulence factors/PBS, encapsulation index and PO activity, with the homogeneity of variance. Welch’s/Brown-Forsythe ANOVA and Dunnett’s T3 multiple comparisons test was used to reveal the influence of virulence on host THCs at 24 h post injection. Unpaired *t*-test (*p* < 0.05) was used to determine significant differences of THCs between the two hosts. The above statistical analyses were conducted using GraphPad Prism 8.0.2 for Windows. The DHC ratio and phagocytosis ratio among treatments were analysed by R × C *chi*-square test at statistical significance of *p* < 0.01 using SPSS 22.0 (IBM Corp. Armonk, NY, USA).

## 5. Conclusions

The venom and ovarian fluids of *T. brontispae* contribute to the success of parasitism on hosts, and the venom is essential for adaptability to a new host. The venom of *T. brontispae* unloaded weapons-granulocytes to inhibit host immunity and decompose/degrade the host fat body, having a stronger virulence in the case of Tb-Onvenom. Thus, *T. brontispae* generated stress-induced evolutionary adaptations in *O. nipae,* which made *O. nipae* a perfect alternative host. *T. brontispae* performed well when encountering local versus non-local hosts, suggesting that the parasitoid wasp has good evolutionary plasticity and that the theory of local adaptation does not apply to this system. The lack of local adaptation can be explained by a number of reasons due to the complexity of the traits involved in the host-parasitoid interactions and/or strategies used by *T. brontispae*. Our findings suggest that the venom undergoes rapid evolution when facing different hosts, which helps to expand the host range. We presume that the *T. brontispae*-*B. longissima*/*O. nipae* systems are useful models for understanding of the evolutionary processes of the parasitoid wasps targeting different hosts with variable physiological dimensions, illustrating the trade-off mechanisms in parasitoid strategies. A better understanding of the functions of venom composition, host immune response traits, hormone system, nutrition metabolism and participating genes is required to further investigate the mechanism of stress-induced evolution and trade-off in the parasitoid wasp and host system.

## Data Availability

The data that support the findings of this study are available from the corresponding author upon reasonable request.

## Supplementary Materials

The following are available online at https://www.mdpi.com/article/10.3390/ijms22073581/s1.
