# Supplementary material for: Variation in Parasitoid Virulence of Tetrastichus brontispae during the Targeting of Two Host Beetles
Source: Int J Mol Sci. 2021 Mar 30;22(7):3581. doi: 10.3390/ijms22073581 (PMC8036858; doi:10.3390/ijms22073581)
Supplement: Supplementary file 1 [file ijms-22-03581-s001.pdf]

Supplementary Materials

Table S1. Assembly summary of the *O. nipae* and *B. longissima* transcriptomes.

**Sequencing Parameters**	**Number**	
	O. nipae	***B. longissima***
Total number	42795	31145
Total base	45693461	38840225
Largest length (bp)	36829	21223
Smallest length (bp)	201	201
Average length (bp)	1067.73	1021.14
N50 length (bp)	2037	1829
E90N50 length (bp)	3557	2258
GC percent (%)	35.21	39.91
TransRate score	0.25263	0.37534
BUSCO score	78.0% (3.2%)	86.9% (5.6%)

De novo assembly produced 42,795 and 31,145 unigenes in *O. nipae* and *B. longissima* with an average length of 1067 and 1021 bp, respectively.

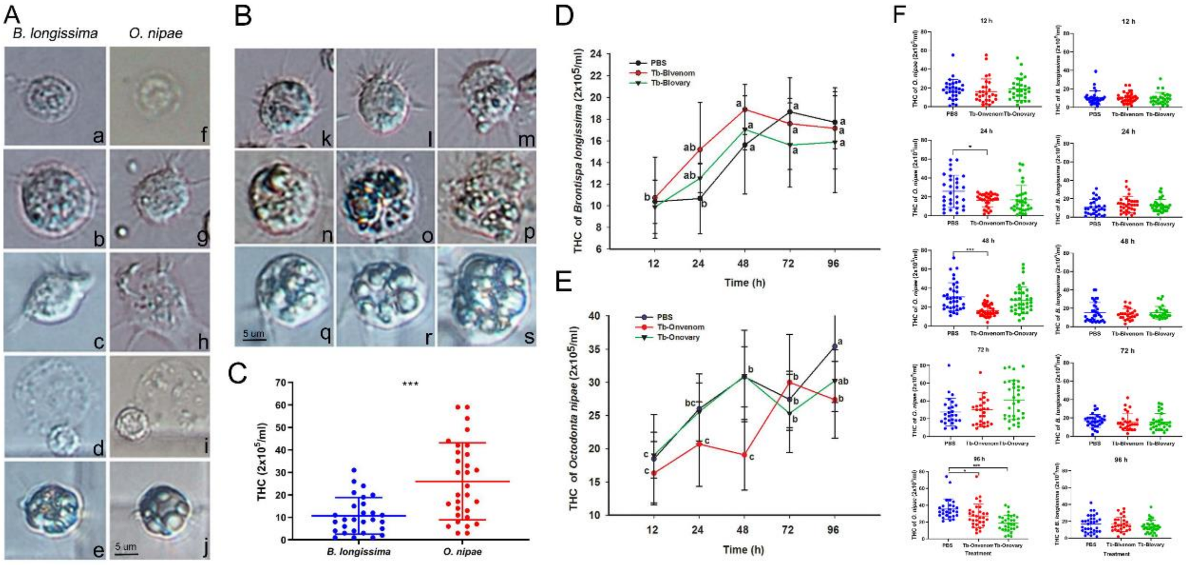

Figure S1. Comparison of cytotoxicity of venom or ovarian fluids injection against *O. nipae* and *B. longissima* pupae. (**A**) Morphology of haemocytes from both beetles determined by differential interference microscopy. **a, f** prohaemocytes (Ph); **b, g** granulocytes (Gr); **c, h** plasmatocytes (Pl); **d, i** oenocytoids (Oe); **e, j** spherulocytes (Sp). Scale bars, 5 μm. (**B**) Polymorphism of granulocytes, **k, l, m** normal extension of granulocytes; **n-s** Macrogranulocytes (MGrs) with more abundant and larger granules in their cytoplasm are the activated granulocytes with ruptures at the end. (**C**) Comparison of THCs between *O. nipae* and *B. longissima.* (**D**) THCs of *B. longissima* at various time points after the injection with Tb-Blvenom (red) or Tb-Blovary (green). (**E, F**) THCs of *O. nipae* at various time points after the injection with Tb-Onvenom (red) or Tb-Onovary (green). *** in (**C**)represents a significant difference at the $p < 0.001$ level (unpaired t test). Different letters in (**D**) and (**E**) show a significant difference at the 0.05 level among different time points (one-way ANOVA followed by Dunnett's test). * and ** in (**F**) represent a significant difference at the $p < 0.05$ level and $p < 0.01$ level, respectively (Welch's/Brown-Forsythe ANOVA followed by Dunnett's T3 test).

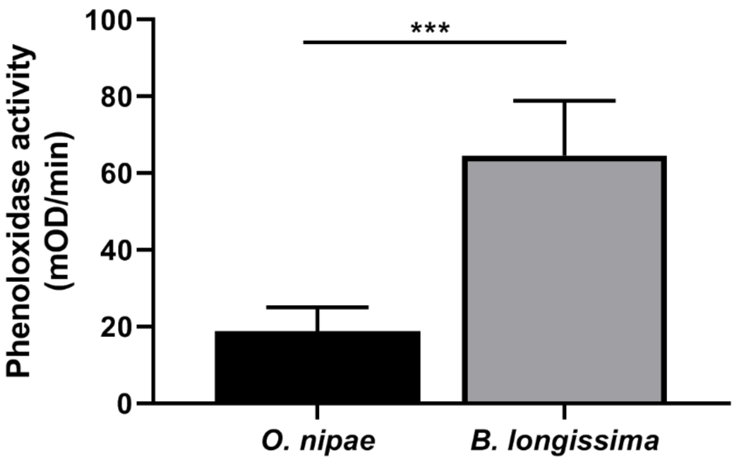

Figure S2. Phenoloxidase activity of *O. nipae* and *B. longissima* pupae. Haemolymph was collected from the *O. nipae* and *B. longissima* pupae 24 h after PBS injection. *** represents a significant difference at the $p < 0.001$ level (unpaired *t* test).

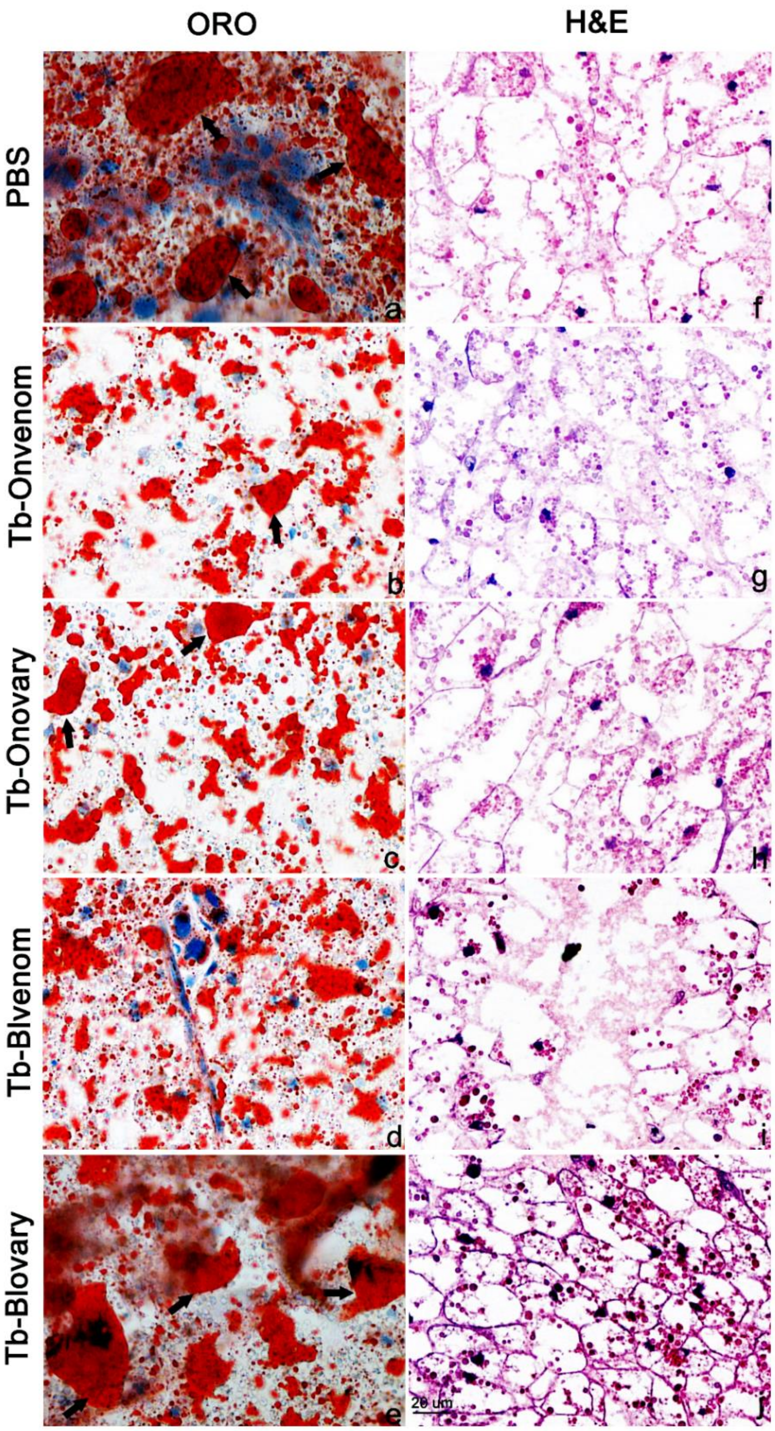

Figure S3. Effect of virulent factors on *O. nipae* pupae fat body lysis 24 h post injection. ORO (left) and H&E (right) stained fat body tissues of *O. nipae* pupae injected with PBS (**a, f**), Tb-Onvenom (**b, g**), Tb-Onovary (**c, h**), Tb-Blvenom (**d, i**) or Tb-Blovary (**e, j**); original magnification, 40×. Larger piece of fat body is marked by thick black arrows in the ORO sections. Scale bars, 20 μm.

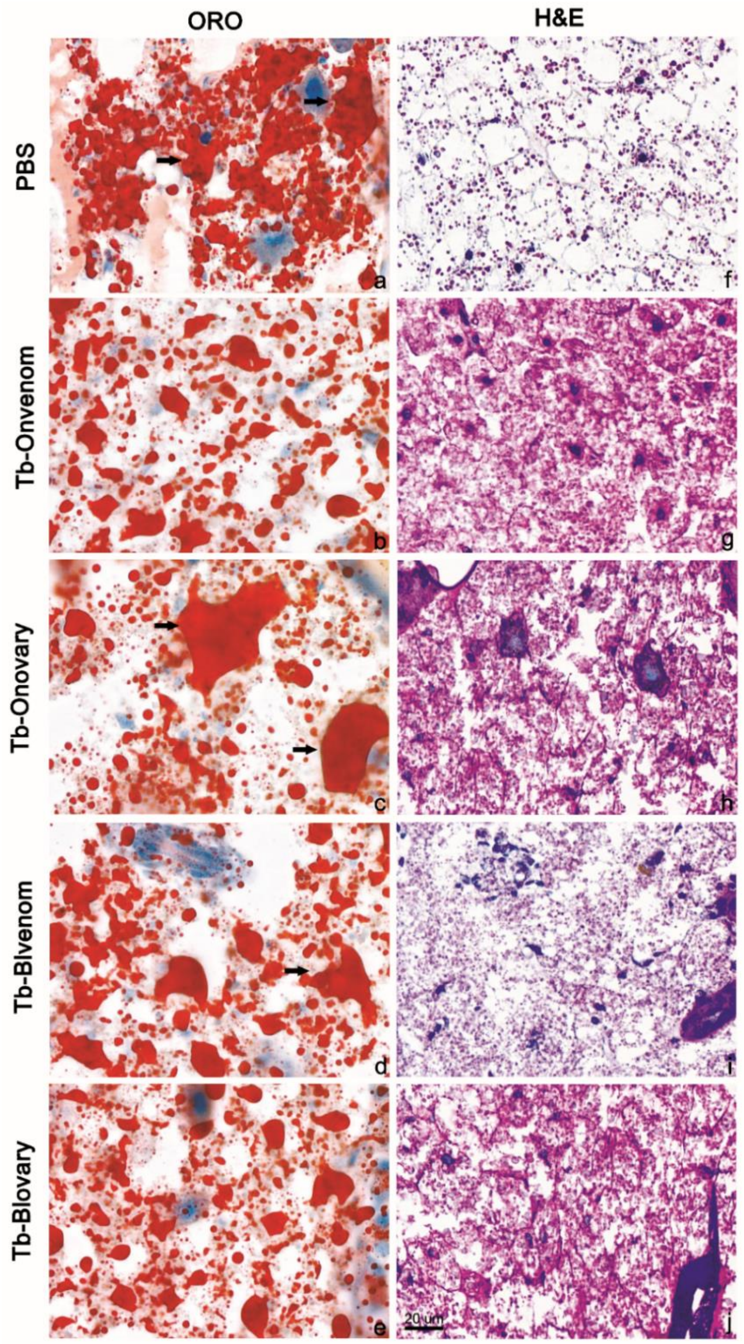

Figure S4. Effect of virulent factors on *B. longissima* pupae fat body lysis 48 h post injection. ORO (left) and H&E (right) stained fat body tissues of *B. longissima* pupae injected with PBS (**a, f**), Tb-Onvenom (**b, g**), Tb-Onovary (**c, h**), Tb-Blvenom (**d, i**) or Tb-Blovary (**e, j**); original magnification, 40x. Larger piece of fat body is marked by thick black arrows in the ORO sections. Scale bars, 20 µm.

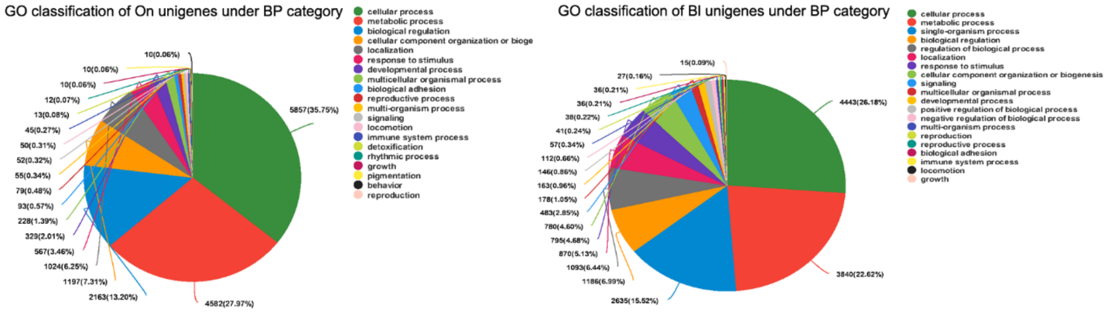

Figure S5. GO classification of *O. nipae* or *B. longissima* unigenes under Biological Process (BP) category.

GO analysis of the biological process category indicated that the top three groups were cellular process (5857, 35.75%), metabolic process (4582, 27.97%) and biological regulation (2863, 13.20%) in *O. nipae* and cellular process (4443, 26.18%), metabolic process (3840, 22.62%) and single-organism process (2635, 15.25%) in *B. longissima* (Figure S5).

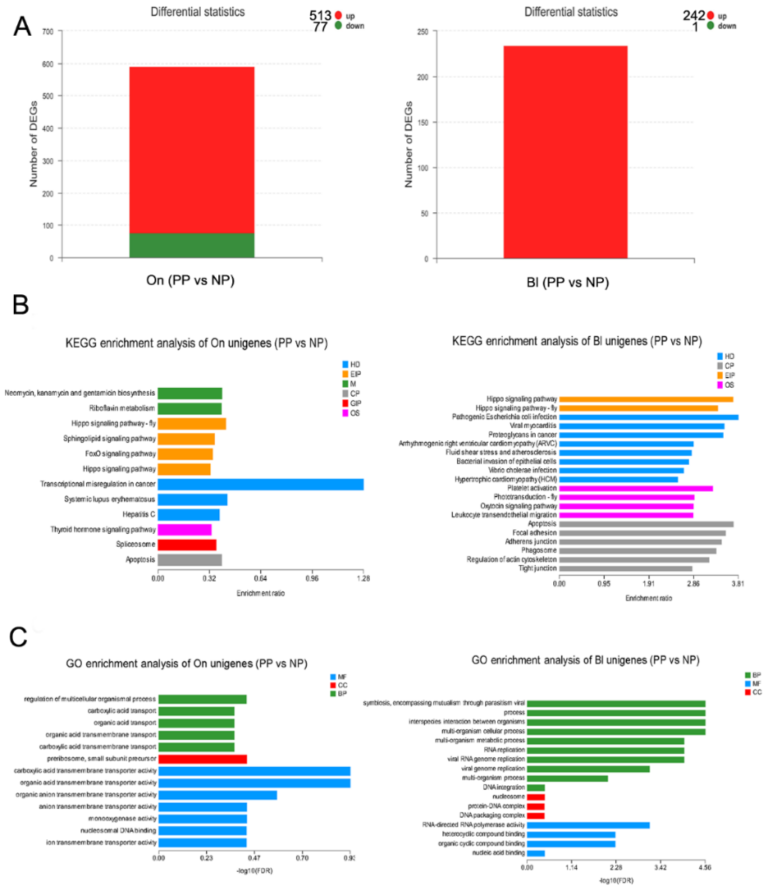

Figure S6. Comparative transcriptomic analysis of *O. nipae* or *B. longissima* parasitized by *T. brontispae*. (**A**) Differential expressed genes (DEGs) between non-parasitized and parasitized pupae. (**B, C**) Enrichment analysis of DEGs. HD, human disease; EIP, environmental information processing; M, metabolism; CP, cellular processes; GIP, genetic information processing; OS, organismal systems. MF, molecular function; CC, cellular component; BP, biological process.

Using KEGG enrichment analysis, most of the DEGs in *O. nipae* were categorized into the pathways of environmental information processing (EIP) and genetic information processing (GIP) (Figure S6B); in *B. longissima*, the majority of the DEGs were categorized into the pathways of human disease (HD) and cell processing (CP) (Figure S6B). GO enrichment analysis showed that the enriched terms for DEGs in *O. nipae* were completely different from that in *B. longissima* (Figure S6C).
